# Clinical outcomes after surgery for benign stomach versus colorectal perforations

**DOI:** 10.1186/s12893-025-03338-1

**Published:** 2025-12-05

**Authors:** Saleh Lahes, Nour Alkhanji, Iurii Snopok, Mohammed Abdulla, Gudrun Wagenpfeil, Matthias Glanemann

**Affiliations:** 1 Surgical Clinic, University Hospital Knappschaftkliniken Bochum, Ruhr University Bochum, In der Schornau 23-25, Bochum, 44892 Germany; 2 Institute for Medical Biometrics, Epidemiology and Medical Computer Science, Saarland University, Homburg/Saar, Germany; 3 Department of General, Visceral, Vascular and Pediatric Surgery, Saarland University, Homburg/Saar, Germany

**Keywords:** Stomach perforation, Colorectal perforation, Clinical outcome, Mortality

## Abstract

**Background:**

Free perforation of stomach or colorectum usually necessitates emergency surgery. Perforation is associated with short-term mortality and morbidity in up to 30% and 50% of patients, respectively, due to secondary peritonitis and sepsis. We hypothesized that postoperative clinical outcomes after colorectal perforation (CRP) are worse than those with stomach perforation (SP). This retrospective study aimed to compare the early postoperative clinical outcomes of patients with stomach and colorectal perforations, focusing on morbidity and mortality, and identify differences that could indicate potential changes in surgical management.

**Methods:**

Data from 214 patients who underwent surgery for stomach and colorectal perforations were retrospectively reviewed.

We compared the demographic, intraoperative, and postoperative data, including morbidity and mortality during hospitalization of the two groups of patients. One group consisted of patients with SP, and the second group consisted of patients with CRP.

**Results:**

The incidences of postoperative cardiac, gastrointestinal, and nephrological complications; sepsis; planned reoperation; reoperation due to complications; median operation time; median blood loss; and median hospital stay after surgery were significantly higher in patients with CRP than in those with SP. After adjusting for potential confounders such as BMI and nicotine use using a logistic regression model, we observed additional that the incidence of postoperative surgical complications was significantly higher in the CRP group compared to the SP group. Peritonitis was significantly more common in the SP group. In addition, overall medical complications and mortality were common in the total patient population and were higher in the CRP than in SP group; however, these differences were not statistically significant.

**Conclusion:**

Mortality and morbidity rates following surgery for SP and CRP were similar and often life threatening. However, patients with CRP experience worse clinical outcomes more often, indicating a more complex injury requiring considerably more medical intervention and extended treatment.

## Introduction

Stomach perforation (SP) most commonly results from peptic ulcer disease but may also occur due to trauma, malignancies, interventional procedures, or intrinsic stomach pathologies. In rare cases, it can develop spontaneously, particularly in newborns [1, 2]. Patients with SP may present with mild localized pain or signs of infection, including peritonitis or sepsis, as the worst clinical presentation. SP is the second most common cause of abdominal perforation and the primary reason for emergency stomach surgery. Perforation can also lead to peritonitis and sepsis. Rapid diagnosis and treatment are required to reduce the risk of mortality and sepsis. SP can lead to short-term mortality and morbidity rates of up to 30% and 50%, respectively, and requires rapid and intensive treatment [3].

Colorectal perforation (CRP) can occur as a consequence of various colorectal diseases or during colonoscopy procedures. Disease-related perforations include appendicitis, diverticulitis, or colorectal cancer. Colon perforation can also occur because of a toxic megacolon associated with ulcerative colitis or *Clostridium* colitis.

Sepsis or septic shock following CRP continues to be associated with high mortality rates, ranging from 6% to 33% [4–7]. Fecal peritonitis leads to complications causing multiple organ failure and sepsis and is one of the reasons for the high mortality rate.

In-hospital mortality in patients with CRP was related to several variables such as age, preoperative septic shock, concomitant disabling cardiac disease, concomitant end-stage renal failure, low preoperative platelet count, and peritonitis [7–9].

Surgical treatment for gastrointestinal perforation is urgently needed, and preoperative knowledge of the severity of the perforation and associated risk factors is essential.

Patients usually require intensive care following surgery and often have prolonged hospitalization. We hypothesized that postoperative clinical outcomes after CRP are worse than those with SP.

This study aimed to identify clinically relevant prognostic factors for patients with stomach and colorectal perforations and to compare the clinical outcomes, which could be positively influenced by any change in surgical management to improve patient outcomes.

## Patients and methods

This study was conducted at the University Hospital of Saarland between 2012 and 2022 to evaluate patients with SP or CRP who had undergone abdominal surgery. The study included patients with free perforation of the stomach or colorectum caused by ulcer, diverticulitis, ischemia, colitis, trauma, bleeding, ileus, hernia, cholecystocolonic fistula and vasculitis but not those with perforation caused by endoscopy or abdominal surgery. Furthermore, patients with malignancy-related perforations were excluded (Table 3).

All patients underwent emergency surgery. The time interval between diagnosis and surgery ranged from 30 min to 4 h. All patients received antibiotics both during and after the surgery.

Demographic data and preoperative information were extracted from the patient charts, including sex, age, body mass index (BMI), American Society of Anesthesiologists (ASA) score, Charlson Comorbidity Index (CCI), previous abdominal surgery, and nicotine use (Table 1). The duration of surgery, intraoperative blood loss, presence of peritonitis, and localization of the perforation were recorded (Tables 1 and 2). Table 3 lists the reasons of perforation.

**Table 1:** Perioperative data and patient characteristics

	Stomach Perforation (SP) *n* = 74	Colorectal Perforation (CRP) *n* = 140	Overall *n* = 214	Univariate Analysis *p*-value for comparison of SP and CRP
Male/female ratio	42/32	71/69	113/101	0.472
Median Age (years) range	64 (19–96)	70 (23–93)	69.5 (19–96)	0.238
BMI, median, range	23.7 (15–38.1)	27.5 (15.6–49.2)	26.2 (15–49.2.2)	< 0.001
ASA				0.152
I	3 (4.1%)	1 (0.7%)	4 (1.9%)	
II	10 (13.5%)	22 (15.7%)	32 (15%)	
III	39 (52.7%)	58 (41.4%)	97 (45.3%)	
IV	21 (28.4%)	59 (42.1%)	80 (37.4%)	
V	1 (1.4%)	0 (0%)	1 (0.5%)	
Charlson Comorbidity Index (CCI)				0.753
0	0	6 (4.3%)	6 (2.8%)	
1	10 (13.5%)	13 (9.3%)	23 (10.7)	
2	7 (9.5%)	6 (4.3%)	13 (6.1%)	
3	11 (14.9%)	13 (9.3%)	24 (11.2%)	
4	2 (2.7%)	19 (13.6%)	21 (9.8%)	
5	10 (13.5%)	13 (56.5%)	23 (10.7%)	
6	11 (14.9%)	16 (11.4%)	27 (12.6)	
7	2 (2.7%)	16 (11.4%)	18 (8.4%)	
8	3 (4.1%)	19 (13.6%)	22 (10.3%)	
9	8 (10.8%)	5 (3.6%)	13 (6.1%)	
10	4 (5.4%)	2 (1.4%)	6 (2.8%)	
11	2 (2.7%)	5 (3.6%)	7 (3.3%)	
12	0 (0%)	3 (2.1%)	3 (1.4%)	
13	3 (4.1%)	3 (2.1%)	6 (2.8%)	
14	1 (1.4%)	1 (0.7%)	2 (0.9%)	
Previous abdominal surgery				0.746
Yes	21 (28.4%)	36 (25.7%)	57 (26.6%)	
No	53 (71.6%)	104 (66.2%)	157 (73.4%)	
Nicotine use				0.047
Yes	25 (33.8%)	29 (20.7%)	54 (25.2%)	
No	49 (66.2%)	111 (79.3%)	160 (74.8%)	
Median duration of surgery (min), range	92 (37–292)	127.5 (43–242)	116 (37–292)	< 0.001
Median blood loss in ml (range)	27 (0–3500)	100 (5–2000)	100 (0–3500)	< 0.001
Peritonitis	71 (95.9%)	117 (83.6%)	188 (87.9%)	0.014

Univariate analysis was performed using Pearson‘s chi-square test and Mann-Whitney U tests. Categorical data are presented as absolute numbers and percentages

**Table 2:** Localization of stomach (*n* = 74) or colorectal (*n* = 140) perforation

Stomach:	
Cardia	1 (1.4%)
Fundus	1 (1.4%)
Corpus	11 (14.9%)
Antrum	25 (33.8)
Pylorus	36 (48.6%)

Colorectal:	
Cecum	12 (8.6%)
Colon ascendens	4 (2.9%)
Colon transversum	7 (5%)
Colon descendens	6 (4.3%)
Colon sigmoideum	109 (77.9%)
Rectum	2 (1.4)

**Table 3:** Cause for perforation

Stomach perforation *n*:74	Colorectal perforation *n*:140
Ulcer 74 (100%)	Diverticulitis 108 (77.4%)
	Ischemia 15 (10.7%)
	Colitis 8 (5.7%)
	Trauma 2 (1.4%)
	Ulcer 2 (1.4%)
	Bleeding 1 (1,7%)
	Ileus 1 (1,7%)
	hernia 1 (1,7%)
	cholecystocolonic fistula 1 (1,7%)
	vasculitis 1 (1,7%)

Surgical procedures included ulcer excision and suturing, gastric resection, colon resection, and other reconstructive procedures as required (Table 4). In more than 98% of cases, the procedures were performed using laparotomy.

**Table 4:** Surgical procedures

Stomach perforation	*n*, 74 (100%)
Ulcer excision and suture	67 (90.5%)
External biliary diversion via T-tube	4 (5.4%)
Antrum resection and Roux-en-Y gastrojejunostomy	4 (5.4%)
Wedge resection	2 (2.7%)
Gastrectomy	1 (1.4%)

Colorectal perforation	*n*, 140 (100%)
Sigmoid resection (Hartmann's procedure)	72 (51.4%)
Anterior rectum resection (Hartmann's procedure)	21 (15%)
Hemicolectomy left (Hartmann's procedure)	11 (7.9%)
Hemicolectomy right with anastomosis	10 (7.1%)
Sigmoid resection with anastomosis	6 (4.3%)
Hemicolectomy right with stoma	4 (2.8%)
Colon segment resection with anastomosis	4 (2.8%)
Anterior rectum resection with anastomosis	3 (2.1%)
Subtotal colectomy with ileostomy (Hartmann's procedure)	3 (2.1%)
Ileocecal resection with anastomosis	2 (1.4%)
ileocecal resection with stoma	2 (1.4%)
Colon transverse resection (Hartmann's procedure)	1 (0.7%)
Hemicolectomy left with anastomosis	1 (0.7%)

The 30-day postoperative period was recorded and characterized as a normal postoperative course if no complications occurred. Surgical complications, medical complications, sepsis, mortality, duration of hospital stay, and reoperation rate were recorded (Table 5).

**Table 5:** Postoperative complications and clinical outcomes

	Stomach Perforation (SP) *n* = 74	Colorectal Perforation (CRP) *n* = 140	Overall *n* = 214	Univariate Analysis *p*-value for comparison of SP and CRP	Multivariate Analysis (adjusted for BMI and nicotine use) *p*-value for comparison of SP and CRP
No complication	33 (44.6%)	44 (31.4%)	77 (36%)	0.072	OR = 0,593; 95% CI [0,323-1.087.087.087.087]; *p* = 0.091
Surgical complications	24 (32.4%)	63 (45%)	87 (40.7%)	0.081	OR = 1,988; 95% CI [1.065–3.710]; *p* = 0.031
Wound infection	20 (27%)	52 (37.1%)	72 (33.6%)	0.171	OR = 1.869; 95% CI [0.972–3.595]; *p* = 0.061
Suture leak	1 (1.4%)	10 (7.1%)	11 (5.1%)	0.102	OR = 6.456; 95% CI [0.778–53.577]; *p* = 0.084
Bleeding	2 (2.7%)	5 (3.6%)	7 (3.3%)	1.000	OR = 1.124; 95% CI [0.2–6.336.2.336]; *p* = 0.894
Other surgical complication (Abscess, fascial dehiscence, compartment, ischemia)	16 (21.6%)	34 (24.3%)	50 (23.4%)	0.736	OR = 1.437; 95% CI [0.703–2.938]; *p* = 0.321
Medical complication	31 (41.9%)	65 (46.4%)	96 (44.9%)	0.565	OR = 1,263; 95% CI [0.695–2.294]; *p* = 0.443
Cardiac	8 (10.8%)	44 (31.4%)	52 (24.3%)	0.001	OR = 3.599; 95% CI (1.557–8.321); *p* = 0.003
Pulmonary	22 (29.7%)	53 (37.9%)	75 (35%)	0.292	OR = 1.520; 95% CI [0.808–2.861]; *p* = 0.194
Gastrointestinal	2 (2.7%)	31 (22.1%)	33 (15.4)	< 0.001	OR = 11.168; 95% CI [2.532–49.252]; *p* = 0.001
Neurological	5 (6.8%)	19 (13.6%)	24 (11.2%)	0.173	OR = 2.056; 95% CI [0.711–5.946]; *p* = 0.183
Nephrological	4 (5.4%)	38 (27.1%)	42 (19.6%)	< 0.001	OR = 5.893; 95% CI [1.977–17.568]; *p* = 0.001
Urological	1(1.4%)	1 (0.7%)	2 (0.9%)	1.000	OR = 0.203; 95% CI [0.009–4.512]; *p* = 0.313
Sepsis	18 (24.3%)	55 (39.3%)	73 (34.1%)	0.034	OR = 1.799; 95% CI [0.935–3.463]; *p* = 0.079
Mortality, n	14 (18.9%)	39 (27.9)	53 (24.8%)	0.183	OR = 1.589; 95% CI [0.775–3.259]; *p* = 0.206
Median duration of postoperative hospital stays (range), days	12.5 (1–77)	23 (1–113)	19 (1–113)	< 0.001	Exp (B) = 1.60; 95% CI [1.28–2.02]; *p* < 0.001
Planned reoperation	4 (5.4%)	44 (31.4%)	48 (22.4%)	< 0.001	OR = 7.823; 95% CI [2.636–23.221]; *p* < 0.001
Reoperation because of complication	17 (23%)	55 (39.3%)	72 (33.6%)	0.022	OR = 2.480; 95% CI [1.264–4.865]; *p* = 0.008

Univariate analysis was performed using Pearson‘s chi-square test and Mann-Whitney U tests. multivariate analysis was conducted using a logistic regression model. The median duration of postoperative hospital stay was modeled using a generalized linear model with a gamma distribution and log link function. Categorical data are presented as absolute numbers and percentages

Statistical analysis was performed using SPSS^®^ version 28.0 (IBM, Armonk, NY, USA). Categorical variables were presented as frequencies and percentages, and compared using the Chi-squared test. Continuous variables were compared using the Mann-Whitney U test. A *p*-value < 0.05 was considered statistically significant. To adjust for potential confounders such as body mass index (BMI) and nicotine use, multivariate analysis was conducted using a logistic regression model. The median duration of postoperative hospital stay was modeled using a generalized linear model with a gamma distribution and log link function.

## Results

A total of 214 patients were included in this study: 74 with SP and 140 with CRP. Among them, 113 were male and 101 were female. All patients underwent emergency surgery. The median age of the cohort was 69.5 years, and the median BMI was 26.2. Most patients had an ASA score of 3 and a CCI of 3.

Demographic characteristics were comparable between the SP and CRP groups, with the exception of BMI and nicotine use (Table 1). Patients in the CRP group had a significantly higher BMI compared to those in the SP group (*p* < 0.001). While the majority of patients did not report nicotine use, nicotine use was significantly more common in the SP group (*p* = 0.047).

The duration of surgery differed significantly between the two groups, reaching a median surgical time of 127.5 min (range 43–242) in the CRP group and 92 min (range 37–292) in the SP group (*p* < 0.001).

Intraoperative blood loss was significantly higher in the CRP group compared to the SP group (*p* < 0.001). Conversely, peritonitis was more frequently observed in the SP group than in the CRP group (*p* = 0.014).

The anatomical distribution of perforations varied between the SP and CRP groups. In the SP group, most perforations were located in the pylorus (48.6%), followed by the antrum (33.8%) and corpus (14.9%). Perforations in the cardia and fundus were rare, with only one case identified in each location.

In the CRP group, the majority of perforations occurred in the sigmoid colon (77.9%). Less frequently affected sites included the caecum (8.6%), transverse colon (5.0%), descending colon (4.3%), ascending colon (2.9%), and rectum (1.4%) (Table 2).

The underlying causes of perforation are summarized in Table 3. All cases of SP were attributed to peptic ulcers. In contrast, the most common cause of CRP was diverticulitis (77.3%), followed by ischemia (10.7%) and colitis (5.7%).

Table 4 summarizes the surgical management strategies employed. In the SP group, ulcer excision followed by primary suturing was performed in 67 patients. Additionally, four patients underwent antrectomy with Roux-en-Y gastrojejunostomy. Other interventions included wedge resection in two cases, gastrectomy in one case, and T-drain placement in four cases.

In contrast, management of CRP predominantly involved resection with stoma formation or anastomosis. The most frequent procedure was sigmoid resection with stoma creation, accounting for 51.4% of cases, followed by anterior rectal resection with stoma in 15% of patients. Overall, a stoma was created in 81.8% of CRP cases, typically via Hartmann’s procedure, while primary anastomosis was performed in 18.2%.

The postoperative courses and complications are listed in Table 5. Patients with SP tended to experience a more uneventful postoperative course compared to those with CRP (44.6% vs. 31.4%), although this difference did not reach statistical significance (*p* = 0.072). Both surgical and medical complications were more frequent in the CRP group than in the SP group, but these differences were not statistically significant in univariate analysis *(p* = 0.081 and *p* = 0.565, respectively).

In univariate analysis, postoperative cardiac, nephrological, and gastrointestinal complications occurred significantly more frequently in the CRP group. After adjusting for potential confounders such as BMI and nicotine use using a logistic regression model, multivariate analysis showed that the incidence of postoperative surgical, cardiac, nephrological, and gastrointestinal complications remained significantly higher in the CRP group compared to the SP group.

In both groups, 73 patients (34.1%) presented with sepsis. Univariate analysis showed that patients with CRP had a significantly higher rate of sepsis compared to those with SP (39.3% vs. 24.3%; *p* = 0.034). However, this association was not significant in the multivariate analysis (OR = 1,799; 95% CI [0.935–3.463], *p* = 0.079).

Fifty-three patients died after surgery for SP or CRP, with an overall mortality rate of 24.8%.

The mortality rate among patients with colorectal perforation (CRP) was higher (27.9%) than that among patients with stomach perforation (SP) (18.9%). However, this difference did not reach statistical significance (*p* = 0.183). Although not statistically significant, the absolute difference of nearly 9% may be clinically relevant and suggests a trend toward higher mortality in CRP patients.

The median duration of hospital stay was 12.5 days (range: 1–77) in the SP group, whereas it was significantly longer in the CRP group, reaching 23 days (range: 1–113) (*p* < 0.001). After adjusting for potential confounders such as BMI and nicotine use, patients in the CRP group had a 1.60 times longer median hospital stay compared to those in the SP group (Exp (B) = 1.60, 95% CI [1.28–2.02]; *p* < 0.001).

Planned reoperations occurred in 31.4% of patients in the CRP group compared to 5.4% in the SP group (*p* < 0.001). Reoperations due to postoperative complications were also significantly more frequent in the CRP group (39.3% vs. 23%, *p* = 0.022). These differences remained statistically significant in the multivariate analysis.

## Discussion

Stomach and colorectal perforations are among the most common causes of bowel perforation and secondary peritonitis. These conditions are serious and require prompt medical intervention. Given the diverse etiologies of perforation, treatment must be tailored to the specific mechanism, location, and severity of the injury, as well as the patient’s clinical presentation. In most cases, emergency surgery is necessary to manage the condition effectively. This study aimed to compare the clinical outcomes of patients who underwent surgical treatment for free stomach versus colorectal perforations.

A comparison of the demographic characteristics between the two groups revealed similar results, except for BMI and nicotine abuse, which were similar to those of other studies reporting stomach and colorectal perforation [9–13].

There was a significant difference in the median duration of surgery between the CRP and SP groups, possibly due to the anatomy of the colorectum. The median duration of surgery in our study was shorter than that reported in other studies [14, 15].

SP typically presents with acute abdominal pain, whereas colonic perforations tend to follow a slower progression course, presenting as secondary bacterial peritonitis or localized abscess formation [16, 17]. The SP group developed peritonitis more frequently than the CRP group (*p* = 0.014), possibly because the stomach fluid has more volume and is more liquid.

Most perforations were located in the distal stomach or the sigmoid regions (Table 2). These results are similar to those of previous studies [11, 14, 15].

Perforations have various etiologies. Peptic ulceration is the most common cause of SP, as demonstrated by several studies. Most cases of CRP in our study were caused by diverticulitis of the colon. Yamamoto et al. reported similar results, whereas malignancy caused most CRPs in other studies [14, 18].

The surgical procedure depends on the size and location of the perforation. Ulcer excision and suturing were the most common procedures in the SP group (90.5%). According to our results, Treuheit et al. sutured 90% of gastroduodenal perforations.

In our study, 77% of patients with colorectal perforation underwent Hartmann’s procedure, reflecting current international practices in the management of colon perforation with generalized peritonitis. This aligns with the 2022 update of the World Society of Emergency Surgery (WSES) guidelines, which recommend Hartmann’s procedure for managing diffuse peritonitis in critically ill patients and in selected patients with multiple comorbidities. The high rate of Hartmann’s procedure in our cohort suggests that the severity of contamination, patient instability, and presence of comorbid conditions strongly influenced the choice of surgical approach [19].

CRP surgery has changed over the last few decades, and primary anastomosis has become more effective in selected patients. Horesh et al. concluded that Hartmann’s procedure may not be the optimal surgical intervention for hemodynamically stable patients with perforated diverticulitis and peritonitis. Primary resection with anastomosis with or without a diverting stoma may be considered in clinically stable patients without significant comorbidities [19, 20].

The incidence of surgical complications was higher in the CRP group than in the SP group, and this association remained significant after adjustment for potential confounders in the multivariate analysis (*p* = 0.031). Wound infection was the most common complication in both groups (33.6%), and the results of other studies were similar [5, 11, 12, 21]. There were no significant differences between the two groups in terms of overall medical complications. Cardiac, gastrointestinal, and nephrological complications were significantly more common in the CRP group than in the SP group.

Sepsis occurred in almost 34% of the patients and was a major cause of death. The CRP group had a significantly higher prevalence of sepsis than the SP group. The reason for this was thought to be localized differences in bacterial types and numbers in the colorectum.

Perforations of the colorectal tract are much less frequent than those of the upper gastrointestinal tract, but CRPs have a higher mortality rate of 15–30% [8–10, 13, 22].

Although the difference in mortality rates between CRP and SP groups did not reach statistical significance in our study (27.9% vs. 18.9%, *p* = 0.183 univariate, *p* = 0.206 multivariate), the absolute difference of nearly 9% may still have important clinical implications. CRP is typically associated with fecal peritonitis, characterized by a higher bacterial load and more severe inflammatory response due to contamination with anaerobic and gram-negative flora such as Bacteroides fragilis and Escherichia coli [23]. In contrast, SP often leads to chemical peritonitis with a comparatively lower initial microbial burden [24]. Moreover, patients with CRP may experience delayed presentation due to more subtle early symptoms, contributing to progression of sepsis and poorer outcomes [5]. These pathophysiological and microbiological differences may partly explain the trend toward higher mortality in the CRP group. While our findings did not achieve statistical significance, they highlight the need for further investigation in larger cohorts to assess whether these trends reflect true differences in clinical outcomes and to optimize management strategies accordingly.

According to previous studies, patients in the CRP group spent more time in the intensive care unit and hospital; additionally, they required second-look laparotomies and reoperations because of treatment complications [14–16].

Our comparative study showed that patients with CRP have increased complications, sepsis, mortality, long length of stay and reoperation rates.

Therefore, patients with free stomach or colorectal perforations should receive special attention. Efforts should be made to avoid the ongoing deleterious inflammatory processes. In addition to early and adequate antibiotic treatment, these patients should undergo a second-look laparotomy in some cases.

This study had several limitations. First, operative and postoperative management was performed by different surgeons and was thus inconsistent in quality. This study’s retrospective design and single-center setting represent additional limitations. Prospective studies are practically impossible because of the emergency status and rarity of the condition. However, larger retrospective multicenter studies might improve the external validity of the results. A further limitation of this study is the involvement of multiple surgeons with varying levels of experience, which may have introduced heterogeneity in surgical decision-making and technique, potentially influencing the outcomes.

Despite these limitations, this is the first study of its kind. No comparative studies of stomach and colorectal perforations have previously been reported in the literature.

## Conclusion

In this study, no statistically significant differences were observed between the stomach perforation (SP) and colorectal perforation (CRP) groups with regard to overall postoperative medical complications or mortality. However, both univariate and multivariate analyses demonstrated that patients in the CRP group exhibited significantly higher rates of specific complications, including cardiac, gastrointestinal, and nephrological events, increased intraoperative blood loss, prolonged operative times, elevated reoperation rates, and extended hospital stays. Notably, surgical complications were significantly more frequent in the CRP group in the multivariate analysis, whereas sepsis was significantly more frequent in the CRP group in the univariate analysis. Although mortality was higher in the CRP group, this difference did not attain statistical significance. These findings indicate a trend toward a more complex clinical course among CRP patients, underscoring the need for heightened clinical vigilance and enhanced resource allocation during the perioperative period.

The results from this study are relevant to acute care teams for rapid diagnosis and to general surgeons for improving intraoperative decision-making. They also provide guidance for ICU teams and hospital administrators in managing postoperative complications and optimizing resource utilization.

## Data Availability

The datasets generated during and/or analyzed during the current study are not publicly available, but are available from the corresponding author on reasonable request.

## Abbreviations

SP: Stomach perforation
CRP: Colorectal perforation
BMI: Body mass index
ASA: American Society of Anesthesiologists score
CCI: Charlson Comorbidity Index

## References

[1] Chen TY, Liu HK, Yang MC, Yang YN, Ko PJ, Su YT, Huang RY, Tsai CC. Neonatal gastric perforation: a report of two cases and a systematic review. Med (Baltim). 2018;97(17):e0369. PMID: 29702982; PMCID: PMC5944554. 10.1097/MD.0000000000010369 PMC5944554 29702982

[2] Hasadia R, Kopelman Y, Olsha O, Alfici R, Ashkenazi I. Short- and long-term outcomes of surgical management of peptic ulcer complications in the era of proton pump inhibitors. Eur J Trauma Emerg Surg. 2018;44(5):795–801. 10.1007/s00068-017-0898-z. Epub 2018 Jan 22. PMID: 29354867. 10.1007/s00068-017-0898-z 29354867

[3] Søreide K, Thorsen K, Harrison EM, Bingener J, Møller MH, Ohene-Yeboah M, Søreide JA. Perforated peptic ulcer. Lancet. 2015;386(10000):1288–98. 10.1016/S0140-6736(15)00276-7. PMID: 26460663; PMCID: PMC4618390. 26460663 10.1016/S0140-6736(15)00276-7 PMC4618390

[4] Alvarez JA, Baldonedo RF, Bear IG, Otero J, Pire G, Alvarez P, Jorge JI. Outcome and prognostic factors of morbidity and mortality in perforated sigmoid diverticulitis. Int Surg. 2009;94(3):240–8. PMID: 20187519. 20187519

[5] Bielecki K, Kamiński P, Klukowski M. Large bowel perforation: morbidity and mortality. Tech Coloproctol. 2002;6(3):177 – 82. 10.1007/s101510200039. PMID: 12525912. 10.1007/s101510200039 12525912

[6] Horiuchi A, Watanabe Y, Doi T, Sato K, Yukumi S, Yoshida M, Yamamoto Y, Sugishita H, Kawachi K. Evaluation of prognostic factors and scoring system in colonic perforation. World J Gastroenterol. 2007;13(23):3228–31. 10.3748/wjg.v13.i23.3228. PMID: 17589902; PMCID: PMC4436609. 17589902 10.3748/wjg.v13.i23.3228 PMC4436609

[7] Shimazaki J, Motohashi G, Nishida K, Ubukata H, Tabuchi T. Postoperative arterial blood lactate level as a mortality marker in patients with colorectal perforation. Int J Colorectal Dis. 2014;29(1):51–5. 10.1007/s00384-013-1738-1. Epub 2013 Jul 12. PMID: 23846515; PMCID: PMC3898377. 23846515 10.1007/s00384-013-1738-1 PMC3898377

[8] Kriwanek S, Armbruster C, Beckerhinn P, Dittrich K. Prognostic factors for survival in colonic perforation. Int J Colorectal Dis. 1994;9(3):158 – 62. 10.1007/BF00290194. PMID: 7814991. 10.1007/BF00290194 7814991

[9] Shinkawa H, Yasuhara H, Naka S, Yanagie H, Nojiri T, Furuya Y, Ariki K, Niwa H. Factors affecting the early mortality of patients with nontraumatic colorectal perforation. Surg Today. 2003;33(1):13 – 7. 10.1007/s005950300002. PMID: 12560901. 10.1007/s005950300002 12560901

[10] Komatsu S, Shimomatsuya T, Nakajima M, Amaya H, Kobuchi T, Shiraishi S, Konishi S, Ono S, Maruhashi K. Prognostic factors and scoring system for survival in colonic perforation. Hepatogastroenterology. 2005;52(63):761–4. PMID: 15966200. 15966200

[11] Treuheit J, Krautz C, Weber GF, Grützmann R, Brunner M. Risk factors for postoperative Morbidity, suture Insufficiency, Re-Surgery and mortality in patients with gastroduodenal perforation. J Clin Med. 2023;12(19):6300. 10.3390/jcm12196300. PMID: 37834943; PMCID: PMC10573308. 37834943 10.3390/jcm12196300 PMC10573308

[12] Ahmed M, Mansoor T, Rab AZ, Rizvi SAA. Risk factors influencing postoperative outcome in patients with perforated peptic ulcer: a prospective cohort study. Eur J Trauma Emerg Surg. 2022;48(1):81–6. 10.1007/s00068-020-01597-6. Epub 2021 Feb 15. PMID: 33590271. 33590271 10.1007/s00068-020-01597-6

[13] Kim JM, Jeong SH, Lee YJ, Park ST, Choi SK, Hong SC, Jung EJ, Ju YT, Jeong CY, Ha WS. Analysis of risk factors for postoperative morbidity in perforated peptic ulcer. J Gastric Cancer. 2012;12(1):26–35. 10.5230/jgc.2012.12.1.26. Epub 2012 Mar 30. PMID: 22500261; PMCID: PMC3319796. 22500261 10.5230/jgc.2012.12.1.26 PMC3319796

[14] Yamamoto T, Kita R, Masui H, Kinoshita H, Sakamoto Y, Okada K, Komori J, Miki A, Uryuhara K, Kobayashi H, Hashida H, Kaihara S, Hosotani R. Prediction of mortality in patients with colorectal perforation based on routinely available parameters: a retrospective study. World J Emerg Surg. 2015;10:24. 10.1186/s13017-015-0020-y. PMID: 26213564; PMCID: PMC4513392. 26213564 10.1186/s13017-015-0020-y PMC4513392

[15] Matsuoka T, Yamamoto R, Matsumura K, Kondo R, Kobayashi K, Lefor AK, Sasaki J, Shinozaki H. Perioperative clinical parameters associated with short-term mortality after colorectal perforation. Eur J Trauma Emerg Surg. 2022;48(4):3017–24. 10.1007/s00068-021-01719-8. Epub 2021 Jun 3. PMID: 34081159; PMCID: PMC8172362. 34081159 10.1007/s00068-021-01719-8 PMC8172362

[16] Langell JT, Mulvihill SJ. Gastrointestinal perforation and the acute abdomen. Med Clin North Am. 2008;92(3):599–625, viii-ix. 10.1016/j.mcna.2007.12.004. PMID: 18387378. 10.1016/j.mcna.2007.12.004 18387378

[17] Saturnino PP, Pinto A, Liguori C, Ponticiello G, Romano L. Role of multidetector computed tomography in the diagnosis of colorectal perforations. Semin Ultrasound CT MR. 2016;37(1):49–53. 10.1053/j.sult.2015.10.007. Epub 2015 Oct 17. PMID: 26827738. 26827738 10.1053/j.sult.2015.10.007

[18] Del Gaizo AJ, Lall C, Allen BC, Leyendecker JR. From esophagus to rectum: a comprehensive review of alimentary tract perforations at computed tomography. Abdom Imaging. 2014;39(4):802 – 23. 10.1007/s00261-014-0110-4. PMID: 24584681. 10.1007/s00261-014-0110-4 24584681

[19] Fugazzola P, Ceresoli M, Coccolini F, Gabrielli F, Puzziello A, Monzani F, Amato B, Sganga G, Sartelli M, Menichetti F, Puglisi GA, Tartaglia D, Carcoforo P, Avenia N, Kluger Y, Paolillo C, Zago M, Leppäniemi A, Tomasoni M, Cobianchi L, Dal Mas F, Improta M, Moore EE, Peitzman AB, Sugrue M, Agnoletti V, Fraga GP, Weber DG, Damaskos D, Abu-Zidan FM, Wani I, Kirkpatrick AW, Pikoulis M, Pararas N, Tan E, Broek RT, Maier RV, Davies RJ, Kashuk J, Shelat VG, Mefire AC, Augustin G, Magnone S, Poiasina E, De Simone B, Chiarugi M, Biffl W, Baiocchi GL, Catena F, Ansaloni L, The WSES/SICG/ACOI/SICUT/AcEMC/SIFIPAC guidelines for diagnosis and treatment of acute left colonic diverticulitis in the elderly. World J Emerg Surg. 2022;17(1):5. 10.1186/s13017-022-00408-0. PMID: 35063008; PMCID: PMC8781436. 10.1186/s13017-022-00408-0 PMC8781436 35063008

[20] Horesh N, Emile SH, Khan SM, Freund MR, Garoufalia Z, Silva-Alvarenga E, Gefen R, Wexner SD. Meta-analysis of randomized clinical trials on Long-term outcomes of surgical treatment of perforated diverticulitis. Ann Surg. 2023;278(5):e966–72. 10.1097/SLA.0000000000005909. Epub 2023 May 30. PMID: 37249187. 37249187 10.1097/SLA.0000000000005909

[21] Loire M, Bridoux V, Mege D, Mathonnet M, Mauvais F, Massonnaud C, Regimbeau JM, Tuech JJ. Long-term outcomes of Hartmann’s procedure versus primary anastomosis for generalized peritonitis due to perforated diverticulitis: follow-up of a prospective multicenter randomized trial (DIVERTI). Int J Colorectal Dis. 2021;36(10):2159–2164. 10.1007/s00384-021-03962-2. Epub 2021 Jun 4. PMID: 34086087. 10.1007/s00384-021-03962-2 34086087

[22] Møller MH, Adamsen S, Wøjdemann M, Møller AM. Perforated peptic ulcer: how to improve outcome? Scand J Gastroenterol. 2009;44(1):15–22. 10.1080/00365520802307997. PMID: 18752147. 10.1080/00365520802307997 18752147

[23] Brook I, Frazier EH. Aerobic and anaerobic microbiology in intra-abdominal infections associated with diverticulitis. J Med Microbiol. 2000;49(9):827–830. 10.1099/0022-1317-49-9-827. PMID: 10966232. 10.1099/0022-1317-49-9-827 10966232

[24] Lopez N, Kobayashi L, Coimbra R. A comprehensive review of abdominal infections. World J Emerg Surg. 2011;6:7. 10.1186/1749-7922-6-7. PMID: 21345232; PMCID: PMC3049134. 21345232 10.1186/1749-7922-6-7 PMC3049134

